# Supplementary material for: Strategies to enable large-scale proteomics for reproducible research
Source: Nat Commun. 2020 Jul 30;11:3793. doi: 10.1038/s41467-020-17641-3 (PMC7393074; doi:10.1038/s41467-020-17641-3)
Supplement: Supplementary file 1 — Supplementary Information [file 41467_2020_17641_MOESM1_ESM.pdf]

Supplementary Information

Strategies to enable large-scale proteomics for reproducible research

Poulos et al.

Supplementary Figures

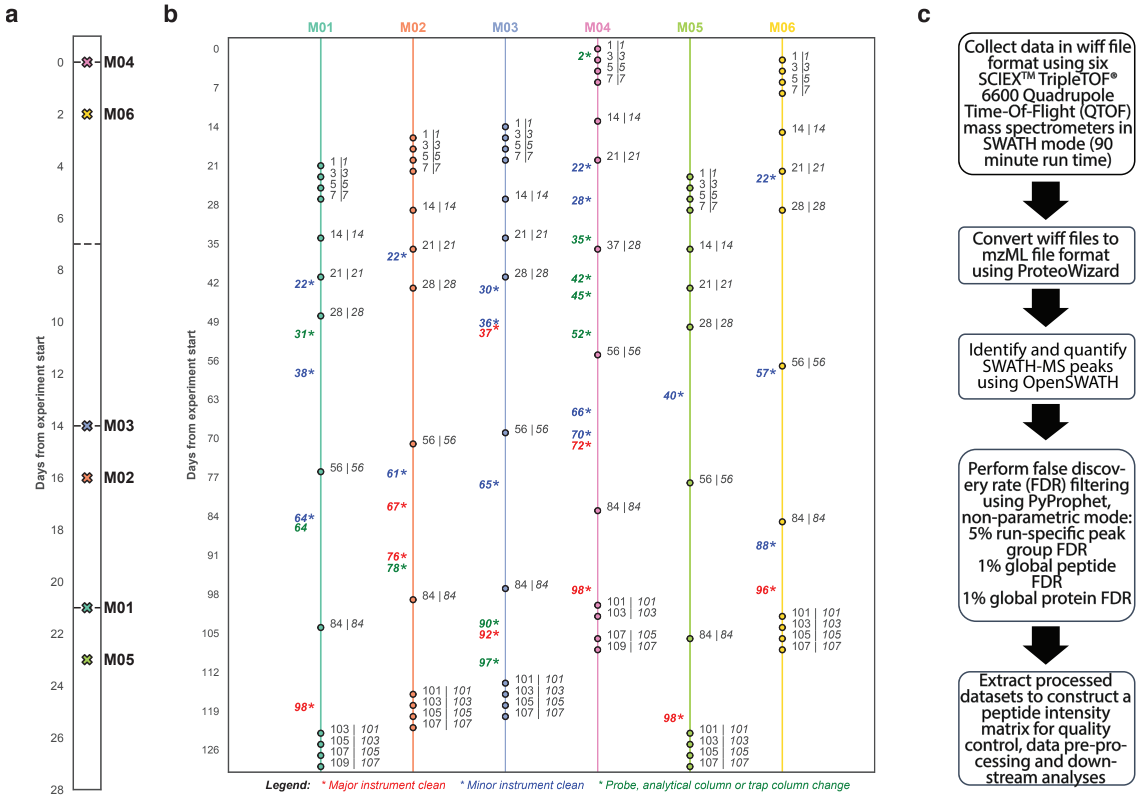

Supplementary Figure 1. Study design and data processing pipeline. (a) Occurrence of experimental *Day 1* for each instrument used in the study. **(b)** Actual timing of data acquisition for this study on each instrument. Data points mark the days for each 48-hour period of data acquisition commenced, relative to the experimental start day. Numbers adjacent to each data point indicate the actual day of data acquisition (left) and the experimental day that it represents in the study design (right, italicised). Experimental days on which instrument maintenance occurred are numbered and indicated by an asterisk. Whenever the instruments were being maintained or not running samples for this study, they were running samples for other studies. **(c)** Flow chart describing the data processing pipeline used to convert raw SWATH-MS data to a final peptide intensity matrix for analysis.

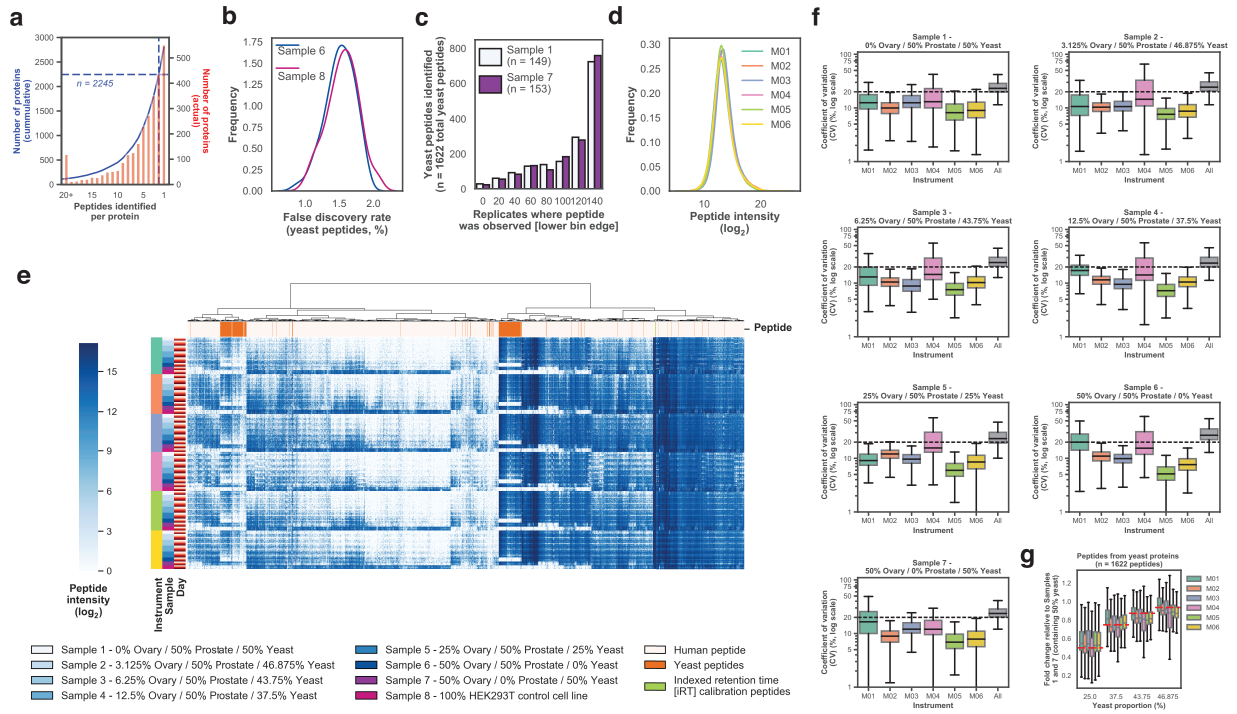

Supplementary Figure 2. Distribution of non-normalised SWATH-MS data. (a) Cumulative (blue; left axis) and actual (red; right axis) numbers of peptides supporting each protein identification. Blue dotted lines indicate the point at which the cumulative number of proteins have support from at least two peptides (n = 2,245 peptides). **(b)** Distribution of experimental false discovery rate (FDR) derived from yeast peptide identifications in replicates of Samples 6 and 8 (containing 0% yeast). **(c)** Numbers of replicates in which each yeast peptide was identified for Samples 1 (n = 149) and 7 (n =153) (containing 50% yeast). **(d)** Distribution of $\log_2$-transformed peptide intensities experiment-wide, coloured by instrument. **(e)** Heatmap of $\log_2$-transformed peptide intensities, ordered on the vertical axis by instrument, sample and then experimental day, respectively. Peptides are clustered on the horizontal axis, with human, yeast and indexed retention time calibration peptides indicated. Missing values are filled with zero. **(f)** Coefficient of variation (CV) per instrument in Samples 1-7. CV was calculated using frequently-observed peptides (n = 2,950 peptides) and using only data acquired during the week after instrument cleaning (days 101, 103, 105 and 107). **(g)** Fold change of the mean of each peptide derived from a yeast protein (n = 1,622 peptides), relative to the mean peptide intensity from Samples 1 and 7 (containing 50% yeast). Only data acquired during the week after instrument cleaning were used and data are shown separately for each instrument. The expected fold change is indicated by a red dashed line. In **(f)** and **(g)**, the box indicates quartiles and the whiskers indicate the rest of the distribution, with outliers not shown. Source data are provided as a Source Data file.

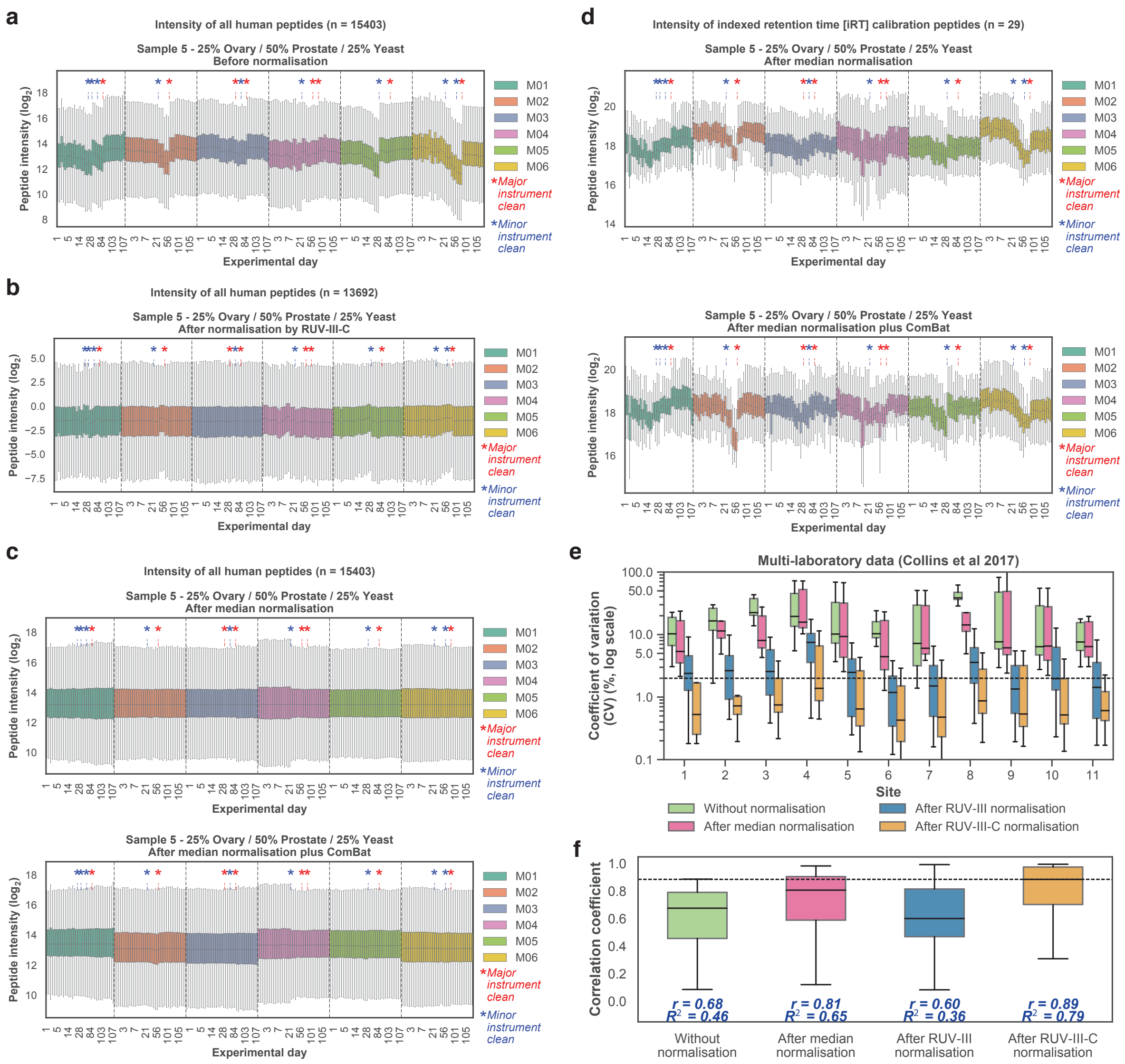

Supplementary Figure 3. Peptide intensity variation during the experimental period following each normalisation approach. (a, b) Intensities of human peptides **(a)** before normalisation (n = 15,4034 peptides) and **(b)** after RUV-III-C normalization (n = 13,692 peptides). **(c, d)** Intensities of **(c)** human peptides (n = 15,403 peptides) and **(d)** indexed retention time calibration peptides (n = 29 peptides) after median normalisation (upper) and after median normalisation plus ComBat (lower). For all plots, boxplots are coloured by instrument, within which data are ordered from earliest experimental day (left) to latest experimental day (right). Maintenance schedules of major (red) and minor (blue) instrument cleaning are indicated. Data are shown for replicates of Sample 5 (containing 25% ovarian cancer tissue / 50% prostate cancer tissue / 25% yeast) and only every sixth experimental day is labelled on the horizontal axis. **(e)** Coefficients of variation (CV) of stable isotope labelled (SIS) peptides (n = 28 peptides) at each experimental site. **(f)** Pearson correlations with the known dilution series of SIS peptides (n = 28 peptides). Median Pearson correlation (*r*) and R^2 from each distribution are shown in italicised blue text. Data shown in **(e)** and **(f)** are from Collins et al.[1] and include data without normalisation and after median, RUV-III and RUV-III-C normalisation. In all plots, the box indicates quartiles and the whiskers indicate the rest of the distribution, with outliers not shown. Source data are provided as a Source Data file.

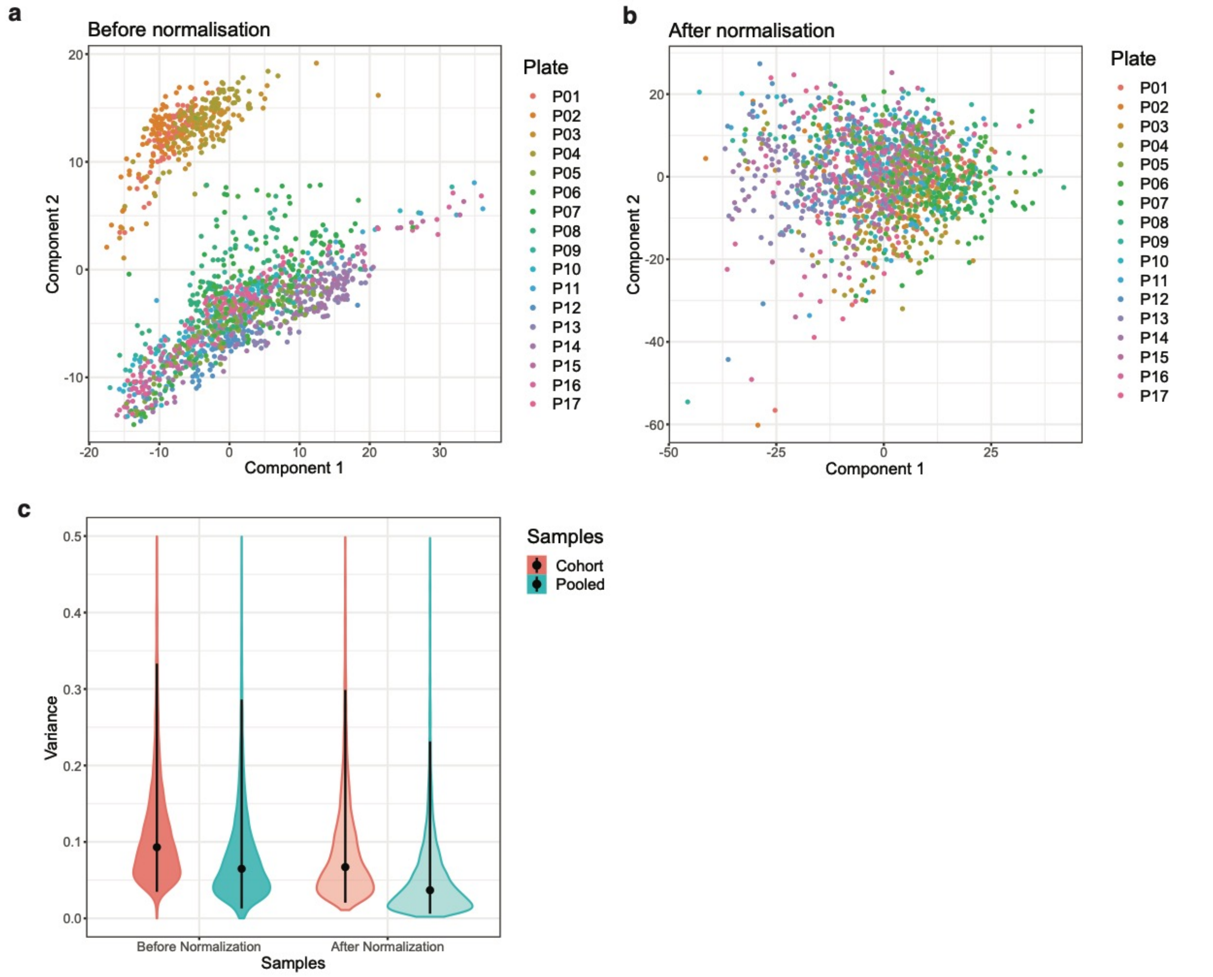

Supplementary Figure 4. Batch effects and variance before and after application of RUV-III-C. **(a, b)** Plots depicting a principal component analysis (PCA) coloured by plate (**a**) before normalisation and **(b)** after normalisation by RUV-III-C. Missing values were replaced with cohort-wide means for the purpose of PCA. **(c)** Variance of pooled replicate samples (red) and the entire cohort of plasma samples (green) before normalisation (leftmost) and after normalisation (rightmost) by RUV-III-C.

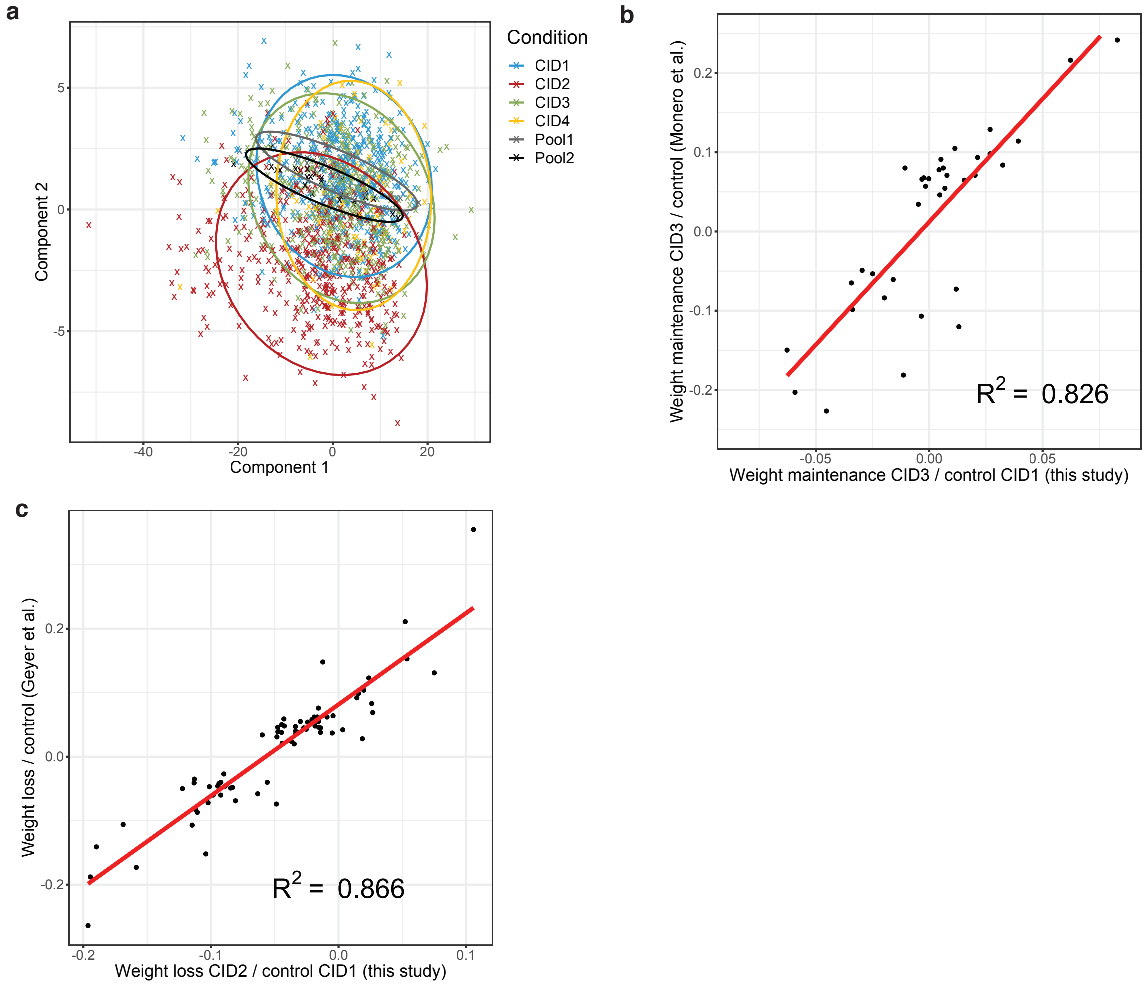

Supplementary Figure 5. Replication of findings from Bruderer et al. (a) Partial least squares discriminant analysis (PLSDA) of the normalised protein data coloured by condition. **(b)** Correlation of the ratios of weight maintenance to baseline from our re-analysis of Bruderer et al. data, against ratios given by Moreno et al. **(c)** Correlation of ratios of weight loss to baseline from our re-analysis of Bruderer et al. data, against ratios given by Geyer et al.

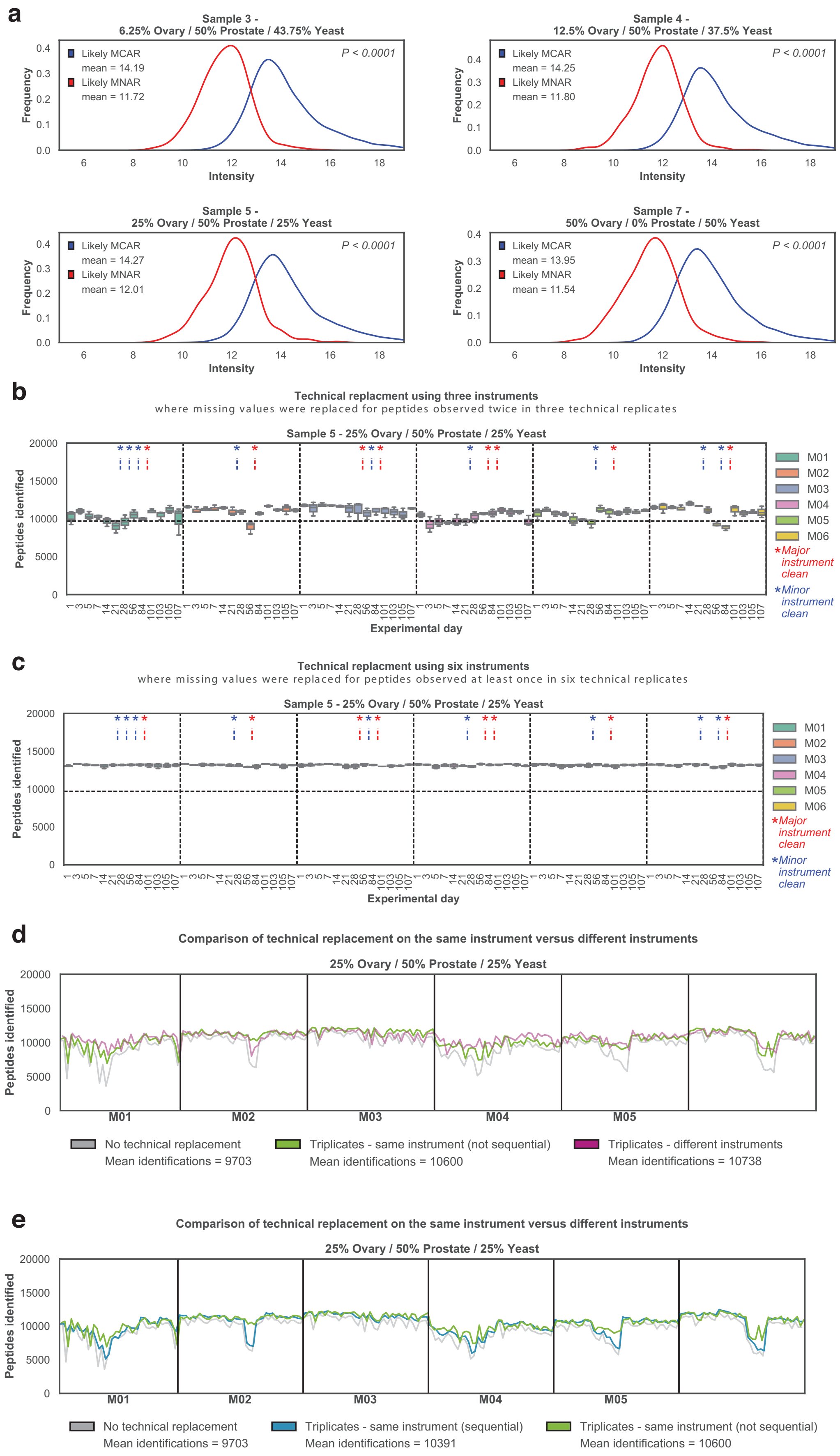
a
Sample 3 - 6.25% Ovary / 50% Prostate / 43.75% Yeast
Likely MCAR mean = 14.19
Likely MNAR mean = 11.72
P < 0.0001
Sample 4 - 12.5% Ovary / 50% Prostate / 37.5% Yeast
Likely MCAR mean = 14.25
Likely MNAR mean = 11.80
P < 0.0001
Sample 5 - 25% Ovary / 50% Prostate / 25% Yeast
Likely MCAR mean = 14.27
Likely MNAR mean = 12.01
P < 0.0001
Sample 7 - 50% Ovary / 0% Prostate / 50% Yeast
Likely MCAR mean = 13.95
Likely MNAR mean = 11.54
P < 0.0001
Frequency
Intensity
b
Technical replacment using three instruments
where missing values were replaced for peptides observed twice in three technical replicates
Sample 5 - 25% Ovary / 50% Prostate / 25% Yeast
Peptides identified
Experimental day
M01
M02
M03
M04
M05
M06
*Major instrument clean
*Minor instrument clean
c
Technical replacment using six instruments
where missing values were replaced for peptides observed at least once in six technical replicates
Sample 5 - 25% Ovary / 50% Prostate / 25% Yeast
Peptides identified
Experimental day
M01
M02
M03
M04
M05
M06
*Major instrument clean
*Minor instrument clean
d
Comparison of technical replacement on the same instrument versus different instruments
25% Ovary / 50% Prostate / 25% Yeast
Peptides identified
M01
M02
M03
M04
M05
No technical replacement
Mean identifications = 9703
Triplicates - same instrument (not sequential)
Mean identifications = 10600
Triplicates - different instruments
Mean identifications = 10738
e
Comparison of technical replacement on the same instrument versus different instruments
25% Ovary / 50% Prostate / 25% Yeast
Peptides identified
M01
M02
M03
M04
M05
No technical replacement
Mean identifications = 9703
Triplicates - same instrument (sequential)
Mean identifications = 10391
Triplicates - same instrument (not sequential)
Mean identifications = 10600

Supplementary Figure 6. Missing values and peptide identifications after technical replacement. (a) Distribution of median non-missing intensity of each peptide designated as likely missing completely at random (MCAR) and missing not at random (MNAR) in Samples 3, 4, 5 and 7. *P*-value determined by two-sided unpaired t-test. **(b, c)** Numbers of peptides identified per experimental day **(b)** after technical replacement using three instruments (where missing values were replaced for peptides observed in two replicates) and **(c)** after technical replacement using six instruments (no constraints on replacement). Boxplots are coloured by instrument, within which data are ordered from earliest experimental day (left) to latest experimental day (right). Maintenance schedules of major (red) and minor (blue) instrument cleaning are also indicated and data are shown for replicates of Sample 5 (containing 25% ovarian cancer tissue / 50% prostate cancer tissue / 25% yeast). The box indicates quartiles and the whiskers indicate the rest of the distribution, with outliers not shown. A horizontal dashed line indicates the mean number of identifications across the experimental period before technical replacement. For replicate numbers n, refer to Supplementary Data 2. **(d, e)** Total numbers of peptides identified per experimental day without technical replacement (grey) and after technical replacement using triplicates measured on the same instrument (sequential: blue; not sequential: green) and different instruments (pink). Data are ordered from earliest experimental day (left) to latest experimental day (right) within the panel for each instrument, and data are shown for the replicates of Sample 5 (containing 25% ovarian cancer tissue / 50% prostate cancer tissue / 25% yeast). Source data are provided as a Source Data file.

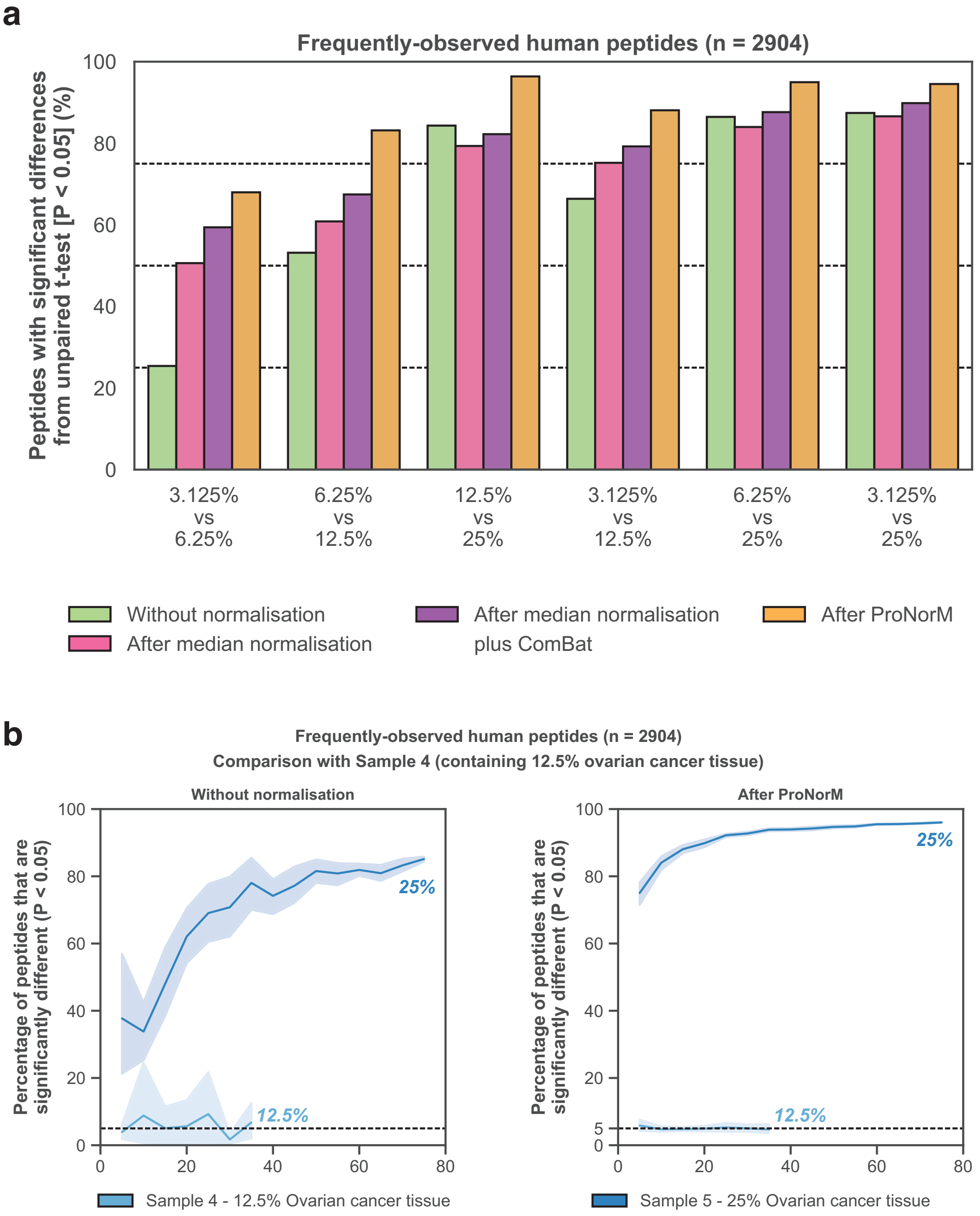

Supplementary Figure 7. Simulation of cohort analyses for discovery proteomics across normalisation methods. (a) Percentage of frequently-observed human peptides (n = 2,904 peptides) found to have significantly different intensities (P < 0.05) between samples with different amounts of ovarian cancer tissue after unpaired two-sided t-test. Data are shown without normalisation, after median normalisation, after median normalisation plus ComBat and after RUV-III-C normalisation. **(b)** Percentage of frequently-observed human peptides that were significantly different (vertical axis) in simulated cohorts of varying sizes (horizontal axis). Plots show comparison between Sample 4 (containing 12.5% ovarian cancer tissue) and Samples 4-5 (containing 12.5% and 25% ovarian cancer tissue), without normalisation (left) and after *ProNorM* (right). Shading denotes 95% confidence intervals derived from ten iterations of random selections of replicates of each sample. For statistical tests in both **(a)** and **(b)**, the mean of each peptide was first calculated within each set of assigned technical triplicates. Source data are provided as a Source Data file.

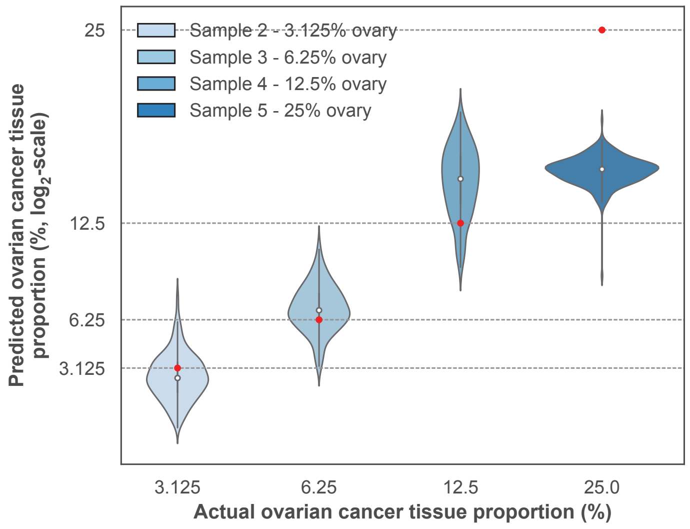

Supplementary Figure 8. Proportion of ovarian cancer tissue predicted by a regularised linear regression model. Violin plots indicate the ovarian cancer tissue proportions predicted by a regularised linear LASSO (Least Absolute Shrinkage and Selection Operator) model. The expected ovarian cancer tissue proportion for each sample is marked by a red data point. Source data are provided as a Source Data file.

Supplementary Tables

A list of iRT peptides

iRT peptides
AGGSSEPVTGLADK
DAVTPADFSEWSK
FLLQFGAQGSPLFK
GDLDAASYYAPVR
GTFIIDPAAIVR
LGGNETQVR
TGFIIDPGGVIR
TPVISGGPYYER
TPVITGAPYYER
VEATFGVDESANK
YILAGVESNK
AAVPSGASTGIYDALELR
ATDAEAEVASLNR
ATDAESEVASLNR
FGVEQNVDMVFASFIR
GDQLFTATEGR
GFLIEGYPR
GILAAEESVGTMGNR
GTGGVDTAAVGAVFDISNADR
LESPDRPFLAILGGAK
LITGEQLGEIYR
LLPSESALLPAPGSPYGR
LQNEVEDLMVDVER
LVSWYDNEFGYSNR
NLAPYSDELR
QVVESAYEVIR
SLEDQLSEIK
SYELPEGQVITIGNER
VLYPNENFFEGK
VVLAYDPVWAIGTGK

Supplementary Table 1. Indexed retention time [iRT] calibration peptides included in each sample before mass spectrometry.

Supplementary Note 1

In Bruderer et al.[2], the authors acquired proteomic data from 1,508 plasma samples via data independent acquisition (DIA) mass spectrometry (MS) using an Orbitrap Fusion Lumos Tribrid mass spectrometer from Thermo Scientific™. Raw data were downloaded according to the published manuscript[2], and we then applied our normalisation approach (RUV-III-C) to the dataset to remove unwanted variation and reproduce essential findings from the published study. Plate-induced batch effects were clearly evident in the raw data (**Supplementary Figure 4a**; see also *Bruderer et al., Supplemental Fig. 4A*).

First, RUV-III-C was applied to the raw transition-level data to normalise the results and remove batch effects. A value of $k = 1$ was used and $n = 706$ negative control variables were selected by using transitions that differed most between batches as measured by a two-sided unpaired t-test. Here we assumed that the most significant variation in these transitions was due to technical variation, with biological variation being comparatively negligible. Replicates were assigned according to the experimental description[2], so that only control samples were replicated. The batch effects were very effectively removed through the application of RUV-III-C (**Supplementary Figure 4b**). After normalisation, the median variance in transition intensities reduced from 0.066 to 0.038 in pooled replicate samples and from 0.095 to 0.061 across the entire cohort of plasma samples (**Supplementary Figure 4c**).

Second, transition-level data were rolled-up to protein-level data using Diffacto[3] [version 1.0.5; default parameters were used with the exception of imputation (no imputation applied) and the minimum number of samples in which a transition must be quantified (100 samples)]. With these protein intensities, we found that the weight loss timepoint (CID2) differed most from both baseline (CID1) and the two time points for weight maintenance (CID 3 and CID4; **Supplementary Figure 5a**). This finding was also reported in Bruderer et al.[2] (see *Bruderer et al., Fig. 4A*).

We next investigated the ratio of intensities at weight maintenance compared to baseline for proteins examined in both Bruderer et al.[2] and Moreno et al.[4] (a previous study that acquired the same samples via data dependent acquisition). Bruderer et al.[2] reported the correlation of ratios between

those from Bruderer et al.[2] and those from Moreno et al.[4], considering only proteins with a consistent effect direction that were significantly different in both studies. When we replicated this finding using data normalised by RUV-III-C, we produced a similarly high R^2 value[2] (**Supplementary Figure 5b** and see *Bruderer et al., Fig. 5A*).

Finally, we investigated the ratio of intensities between baseline and weight loss, for proteins examined in both Bruderer et al.[2] and Geyer et al.[5] (an independent study with a similar design). Bruderer et al.[2] reported the correlation between effect sizes found in Bruderer et al.[2] and those found in Geyer et al.[5]. This finding used only proteins with a consistent effect direction that were significantly different in both studies. When we replicated this finding using data normalised by RUV-III-C, our corresponding value of R^2 was highly similar to that reported in Bruderer et al.[2] (**Supplementary Supplementary Figure 5c** and see *Bruderer et al., Fig. 5B*).

Taken together, these findings demonstrate that our normalisation method of RUV-III-C is applicable to MS measurements acquired on a different DIA-MS instrument platform.

Supplementary Note 2

```
******************************************************************************
--- OpenSWATH ---
# Docker Image = cmriprocan/openms:1.2.4

OpenSwathWorkflow -in /inputs/${rawMzmlSwath} -tr /inputs/${openSwathDecoySrlSql} -tr_irt
/inputs/${openSwathIrtTraml} -out_osw /outputs/${swathScoresSql} -sort_swath_maps -threads 15
-min_upper_edge_dist 1 -readOptions cache -tempDirectory /outputs/.cache -force

******************************************************************************
--- PyProphet ---

----------------- step1 -----------------------
# Docker Image = cmriprocan/openms-toffee:0.14.2

pyprophet subsample --in=/inputs/in.osw --out=/outputs/out.osws --subsample_ratio=$SUB_RATIO

----------------- step2 -----------------------
# Docker Image = cmriprocan/openms-toffee:0.14.2
# Fan-out step

SUBSAMPLED_INPUT_FILES=/inputs/artifacts/out*.osws
TEMPLATE_FILE=/inputs/template.osw  # can be any of the `IN_FILE` from Step 1
OUT_FILE=/outputs/model_scoring.osw

pyprophet merge --out=$OUT_FILE --template=$TEMPLATE_FILE ${SUBSAMPLED_INPUT_FILES}
pyprophet score --no-parametric --in=$OUT_FILE --level=ms1ms2

----------------- step3 -----------------------
# Docker Image = cmriprocan/openms-toffee:0.14.2

IN_FILE=/inputs/out.osw  # Same input file as Step 1
MODEL_FILE=/inputs/model_scoring.osw  # model_scoring.osw from Step 2
OUT_FILE=/outputs/out.oswr  # generates *.oswr files

pyprophet score --no-parametric --in=$IN_FILE --level=ms1ms2 --apply_weights=$MODEL_FILE
pyprophet reduce --in=$IN_FILE --out=$OUT_FILE

----------------- step4 -----------------------
# Docker Image = cmriprocan/openms-toffee:0.14.2
# Fan-out step

REDUCED_INPUT_FILES=/inputs/artifacts/*.oswr
TEMPLATE_MODEL_FILE=/inputs/model_scoring.osw  # model_scoring.osw from Step 2
OUT_FILE=/outputs/model_fdr.osw

pyprophet merge --template=$TEMPLATE_MODEL_FILE --out=$OUT_FILE ${REDUCED_INPUT_FILES}
pyprophet peptide --no-parametric --in=$OUT_FILE --context=global
pyprophet protein --no-parametric --in=$OUT_FILE --context=global

----------------- step5 -----------------------
# Docker Image = cmriprocan/openms-toffee:0.14.2

IN_FILE=/inputs/in.osw  # Same input file as Step 1
MODEL_FILE=/inputs/model_fdr.osw  # model_fdr.osw from Step 4
OUT_FILE="/outputs/out.pyprophet.tsv"

pyprophet backpropagate --in=$IN_FILE --apply_scores=$MODEL_FILE
pyprophet peptide --no-parametric --in=$IN_FILE --context=run-specific
pyprophet protein --no-parametric --in=$IN_FILE --context=run-specific
pyprophet export --in=$IN_FILE --out=$OUT_FILE --format=legacy_merged --
max_global_protein_qvalue=0.01 --max_global_peptide_qvalue=0.01 --
max_rs_peakgroup_qvalue=0.05
```

Supplementary References

1 Collins, B. C. *et al.* Multi-laboratory assessment of reproducibility, qualitative and quantitative performance of SWATH-mass spectrometry. *Nat Commun* **8**, 291 (2017).

2 Bruderer, R. *et al.* Analysis of 1508 Plasma Samples by Capillary-Flow Data-Independent Acquisition Profiles Proteomics of Weight Loss and Maintenance. *Mol Cell Proteom* **18**, 1242-1254 (2019).

3 Zhang, B., Pirmoradian, M., Zubarev, R. & Kall, L. Covariation of Peptide Abundances Accurately Reflects Protein Concentration Differences. *Mol Cell Proteomics* **16**, 936-948 (2017).

4 Oller Moreno, S. *et al.* The differential plasma proteome of obese and overweight individuals undergoing a nutritional weight loss and maintenance intervention. *Proteomics. Clinical applications* **12** (2018).

5 Geyer, P. E. *et al.* Proteomics reveals the effects of sustained weight loss on the human plasma proteome. *Mol Syst Biol* **12**, 901 (2016).
