# Supplementary material for: Strategies to enable large-scale proteomics for reproducible research
Source: Nat Commun. 2020 Jul 30;11:3793. doi: 10.1038/s41467-020-17641-3 (PMC7393074; doi:10.1038/s41467-020-17641-3)
Supplement: Supplementary file 3 — Description of Additional Supplementary Files [file 41467_2020_17641_MOESM3_ESM.docx]

**Description of Additional Supplementary Files**

**Supplementary Data 1**

Details of 1,560 samples generated during this study. M01 indicates instrument number one.

**Supplementary Data 2**

Metadata for each of 1,527 samples analysed in this study. PseudoReplicate indicates the replicate group (numbered) to which the sample was randomly assigned for the purposes of RUV-III-C or technical replacement. M01 indicates instrument number one.
