# Supplementary material for: Strategies to enable large-scale proteomics for reproducible research
Source: Nat Commun. 2020 Jul 30;11:3793. doi: 10.1038/s41467-020-17641-3 (PMC7393074; doi:10.1038/s41467-020-17641-3)
Supplement: Supplementary file 6 — Reporting Summary [file 41467_2020_17641_MOESM6_ESM.pdf]

nature research

Corresponding author(s): Qing Zhong

Last updated by author(s): Jun 9, 2020

Reporting Summary

Nature Research wishes to improve the reproducibility of the work that we publish. This form provides structure for consistency and transparency in reporting. For further information on Nature Research policies, see Authors & Referees and the Editorial Policy Checklist .

Statistics

For all statistical analyses, confirm that the following items are present in the figure legend, table legend, main text, or Methods section.

n/a	Confirmed	
☐	☒	The exact sample size (*n*) for each experimental group/condition, given as a discrete number and unit of measurement
☐	☒	A statement on whether measurements were taken from distinct samples or whether the same sample was measured repeatedly
☐	☒	The statistical test(s) used AND whether they are one- or two-sided<br>*Only common tests should be described solely by name; describe more complex techniques in the Methods section.*
☐	☒	A description of all covariates tested
☐	☒	A description of any assumptions or corrections, such as tests of normality and adjustment for multiple comparisons
☐	☒	A full description of the statistical parameters including central tendency (e.g. means) or other basic estimates (e.g. regression coefficient) AND variation (e.g. standard deviation) or associated estimates of uncertainty (e.g. confidence intervals)
☐	☒	For null hypothesis testing, the test statistic (e.g. *F*, *t*, *r*) with confidence intervals, effect sizes, degrees of freedom and *P* value noted<br>*Give P values as exact values whenever suitable.*
☒	☐	For Bayesian analysis, information on the choice of priors and Markov chain Monte Carlo settings
☒	☐	For hierarchical and complex designs, identification of the appropriate level for tests and full reporting of outcomes
☐	☒	Estimates of effect sizes (e.g. Cohen's *d*, Pearson's *r*), indicating how they were calculated

Our web collection on statistics for biologists contains articles on many of the points above.

Software and code

Policy information about availability of computer code

Data collection	The wiff format files generated in DDA mode were then converted to mgf format using the MSconvert tool from the ProteoWizard40 package [version 3.0.18135 (79c747f66)]. Three search engines, Mascot18 [version 2.6], X!Tandem19 [version 2015.12.15.2] and MSGF+20 [version 2018.09.12] were used for independent searches. Results from Mascot18 (in dat format) , X!Tandem19 (in xml format) and MSGF+20 (in mzid format) were merged using PeptideShaker21 [version 1.16.37]. The merged mzid file was then imported into Skyline22 [version 4.2.0.18305]. Decoy transitions were added by reversing the sequences using OpenSwathDecoyGenerator from msproteomicstools [version 2.4.0-HEAD-2018-12-09]. The library was converted to tsv and TraML formats using OpenSwathAssayGenerator from msproteomicstools to analyse with OpenSwath. The commands used to process SWATH-MS acquisitions through OpenSWATH and PyProphet are recorded in Supplementary File 1. Data files were converted from wiff to mzML format with ProteoWizard [version 3.0.18135 (79c747f66)]. These files were then analysed with OpenSWATH [version 2.4.0, revision a7b4f64], implemented using the Docker container cmriprocan/openms:1.2.4. PyProphet [version 2.0.4] was used for FDR control, implemented using the Docker container cmriprocan/openms-toffee:0.14.2.
Data analysis	Median normalisation was implemented using the NormalyzerDE package [version 1.0.0] in R [version 3.5.2]. ComBat was implemented using the sva package [version 3.30.1] in R [version 3.5.2]. The RUV-III-C implementation is available as an R package stored at the Comprehensive R Archive Network (CRAN).

For manuscripts utilizing custom algorithms or software that are central to the research but not yet described in published literature, software must be made available to editors/reviewers. We strongly encourage code deposition in a community repository (e.g. GitHub). See the Nature Research guidelines for submitting code & software for further information.

Data

Policy information about availability of data

All manuscripts must include a data availability statement. This statement should provide the following information, where applicable:

- Accession codes, unique identifiers, or web links for publicly available datasets
- A list of figures that have associated raw data
- A description of any restrictions on data availability

The mass spectrometry proteomics data including the spectral library, have been deposited to the ProteomeXchange Consortium via the PRIDE52 partner repository with the dataset identifier PXD015912. Source data are provided with this paper. The source data underlying Figs 2b-e, 3b, d-f, 4b-d, 5a-b, 6 and Supplementary Figs 2a-c, f, g, 3e, f, 4b, c, 5a-b and 6 are provided in a Source Data file.

Field-specific reporting

Please select the one below that is the best fit for your research. If you are not sure, read the appropriate sections before making your selection.

[x] Life sciences [ ] Behavioural & social sciences [ ] Ecological, evolutionary & environmental sciences

For a reference copy of the document with all sections, see nature.com/documents/nr-reporting-summary-flat.pdf

Life sciences study design

All studies must disclose on these points even when the disclosure is negative.

Sample size	No sample-size calculation was determined. The sample size was selected to ensure data were collected over a long experimental period (four months) at reasonable intervals within this time frame (13 48-hour time periods). In total, we have run over 1,560 DIA-MS runs on six mass spectrometers operating in a single laboratory over a four-month period, during which approximately 5,000 other samples are also run.
Data exclusions	Data in wiff file format were collected for 1,560 MS runs. Two runs were excluded from downstream analyses due to error during acquisition by the mass spectrometer. One run was removed after OpenSWATH processing due to inability to perform retention time alignment. The data processing pipeline returned data from 1553 runs as no peptides were detectable after PyProphet filtering in four files.
Replication	This study was designed specifically to test the reproducibility of large-scale SWATH-MS experiments with a large number of technical replicates, e.g., n > 100 for each sample.
Randomization	In order to define the matrix M in our present context for RUV-III-C, we modified the notion of 'distinct sample type'. To do so, we randomly partitioned the technical replicates of each of the eight sample types into groups of triplicates acquired on different instruments, and we then declared as technical replicates only those acquisitions corresponding to one of these groups. In effect, we have altered our definition of technical replicates in order to mimic a real-world scenario, whereby we normalise data with RUV-III-C using technical replicates acquired on different instruments at varying times from last clean. For technical replacement, technical replicates were assigned as above but each method used replicates spanning either two, three, four, five or six different instruments respectively.
Blinding	Blinding was not relevant to this study, which was designed to allow the development and evaluation of data analysis and normalization methods for large-scale SWATH-MS experiments. Such methods cannot be developed while blind to the results.

Reporting for specific materials, systems and methods

We require information from authors about some types of materials, experimental systems and methods used in many studies. Here, indicate whether each material, system or method listed is relevant to your study. If you are not sure if a list item applies to your research, read the appropriate section before selecting a response.

Materials & experimental systems

n/a	Involved in the study	
x		Antibodies
	x	Eukaryotic cell lines
x		Palaeontology
x		Animals and other organisms
	x	Human research participants
x		Clinical data

Methods

n/a	Involved in the study	
x		ChIP-seq
x		Flow cytometry
x		MRI-based neuroimaging

Eukaryotic cell lines

Policy information about cell lines

Cell line source(s)	HEK293T cell line was obtained from Cell Bank Australia
Authentication	Cell lines were authenticated by Cell Bank Australian using PCR (Promega kit PCR-PP16HS). Beginning with an authenticated sample, the cells were then expanded and then a bank of several frozen vials was generated for long-term storage. The cultures are generally terminated after ~30 passages.
Mycoplasma contamination	Cultures were regularly checked for mycoplasma contamination; tests were negative.
Commonly misidentified lines (See ICLAC register)	No commonly misidentified cell lines were used in the study.

Human research participants

Policy information about studies involving human research participants

Population characteristics	Ovarian cancer tissue: Ovarian serous carcinoma tissue was obtained from the Gynaecological Oncology Biobank (GynBiobank), Western Sydney Local Health District at Westmead. GynBiobank recruits unselected, consecutive clinic-based donors being investigated for, or diagnosed with gynaecologic cancer.<br>Prostate cancer tissue: Prostate cancer tissues were accessed from the Department of Pathology and Molecular Pathology at the University Hospital Zurich, Switzerland. Covariate relevant population characteristics such as age, gender, genotypic information are irrlevant to this study.<br><br>Population characteristics such as age, gender, genotypic information are not covariates for this study.
Recruitment	Ovarian cancer tissue: Ovarian serous carcinoma tissue was obtained from the Gynaecological Oncology Biobank (GynBiobank), Western Sydney Local Health District at Westmead. GynBiobank recruits unselected, consecutive clinic-based donors being investigated for, or diagnosed with gynaecologic cancer. Most biospecimens are collected at primary surgery.<br>Prostate cancer tissue: Men with clinically localised prostate cancer who were scheduled for radical prostatectomy were selected from a cohort of 1,200 patients within the single-centre Prostate Cancer Outcomes Cohort (ProCOC) study.
Ethics oversight	Ovarian cancer tissue: GynBiobank participants provide written informed consent for the use of biospecimens in research [HREC92/10/4.13].<br>Prostate cancer tissue: The use of prostate cancer tissue was approved by the Cantonal Ethics Committee of Zurich, the associated methods were carried out in accordance with the approved guidelines, and each patient signed an informed consent form [KEK-ZH-No. 2008-0040].<br><br>The use of ovarian and prostate tissue samples in this study was approved by the Western Sydney Local Health District Human Research Ethics Committee [AU RED LNR/16/WMEAD/291 and AU RED HREC/17/WMEAD/63].

Note that full information on the approval of the study protocol must also be provided in the manuscript.
